# Artificial Intelligence in Minimally Invasive Surgery: Current State and Future Challenges

**DOI:** 10.31662/jmaj.2024-0175

**Published:** 2024-11-18

**Authors:** Shintaro Arakaki, Shin Takenaka, Kimimasa Sasaki, Daichi Kitaguchi, Hiro Hasegawa, Nobuyoshi Takeshita, Mitsuhisa Takatsuki, Masaaki Ito

**Affiliations:** 1 Department for the Promotion of Medical Device Innovation, National Cancer Center Hospital East, Kashiwa, Japan; 2 Department of Digestive and General Surgery, Graduate School of Medicine, University of the Ryukyus, Nishihara, Japan; 3 Department of Colorectal Surgery, National Cancer Center Hospital East, Kashiwa, Japan

**Keywords:** Artificial Intelligence, Surgery, Computer Vision, Automated Surgical Skill Evaluation, Machine Learning, Deep Learning, Computer-Assisted Surgery, AICS

## Abstract

Recent advancements in artificial intelligence (AI) have markedly affected various fields, with notable progress in surgery. This study explores the integration of AI in surgery, particularly focusing on minimally invasive surgery (MIS), where high-quality surgical videos provide fertile ground for computer vision (CV) technology applications. CV plays an important role in enhancing intraoperative decision-making through real-time image recognition. This study considers the challenges in clinical applications and future perspectives by reviewing the current state of AI in navigation during surgery, postoperative analysis, and automated surgical skill assessment.

## Introduction

In recent years, the use of artificial intelligence (AI) has increased in various fields, and its development toward practical applications in surgery is also progressing. Minimally invasive surgery (MIS) has led to the accumulation of high-quality surgical videos that serve as valuable medical records with a high affinity for computer vision (CV). CV is a field of AI and computer science that focuses on enabling computers to interpret and understand visual information from digital images or videos, similar to how humans perceive and analyze visual data. It addresses numerous challenges to achieve practical and widespread implementation of these advanced technologies in real-world clinical settings. By delving into current advancements and obstacles faced, this review provides an understanding of the possibilities and limitations of AI in surgery.

## Real-time Support during Surgery

Image recognition AI plays a crucial role in surgeries by providing support through interpreting digital visual information displayed on monitors. This directly aids surgeons in the intraoperative decision-making process. Numerous studies on image recognition AI in surgery have been conducted in recent years ^(1)^.

In laparoscopic cholecystectomy (LC), the YOLOv3 model trained on a dataset of approximately 2000 endoscopic images of the Calot’s triangle region displayed landmarks with the following average precisions: common bile duct 0.320, cystic duct 0.074, lower edge of the left medial liver segment 0.314, and Rouviere’s sulcus 0.101 ^(2)^. During LC, DeepLabv3+ was trained using 1200 images from LapSig300 to display the inferior mesenteric artery. The mean Dice similarity coefficient of the fivefold cross-validation was 0.798 ^(3)^. Similarly, in thoracoscopic esophagectomy, the recurrent laryngeal nerve has been segmented using AI. Forty images extracted from eight thoracoscopic esophagectomy videos were annotated to identify the recurrent laryngeal nerve. The average Dice coefficient of the AI model was 0.58 ^(4)^. In robot-assisted radical prostatectomy, AI segmented the seminal vesicles and the vas deferens. The convolutional neural network model had a Dice similarity coefficient value of 0.73 in the test data ^(5)^.

Although CV for surgery is progressing, current systems find it difficult to recognize structures obscured by substances such as fat or blood. Nevertheless, there is still some significance in recognizing objects already visible on the screen. For example, it has been reported that the major cause of bile duct injury, which is one of the most serious complications in LC, is misrecognition of anatomy where structures such as the common bile duct that should be preserved are erroneously identified as the cystic duct or cystic artery to be dissected. This demonstrates that merely having objects visible within the frame does not necessarily prevent intraoperative adverse events ^(6), (7)^.

Although these systems recognize organs and display their locations, AI models that provide surgeons with instructions for optimal surgical techniques have rarely been reported. Madani et al. assessed the effectiveness of AI models in identifying safe and dangerous zones of dissection and anatomical landmarks during LC ^(8)^. A total of 2627 frames were extracted to annotate the Go and No-Go zones. For the entire dataset, the mean intersection over union and the F1 scores were >0.5 and >0.7, respectively, showing good spatial overlap compared to the ground truths. The accuracy of pixel-wise identification was consistently greater than 90% for all structures ^(8)^. The Society of American Gastrointestinal and Endoscopic Surgeons promotes a Critical View of Safety (CVS) challenge using a different approach, the ^(9)^. CVS is a surgical view to clearly expose the anatomical structure of Calot’s triangle. CVS is recommended to avoid surgical complications, such as bile duct and artery injury ^(10)^. The CVS challenge aims not only to segment landmarks but also to evaluate the CVS during LC ^(9)^. The AI-based CVS assessment achieved accuracy rates of 93.1% for the cystic artery and cystic duct, 68.3% for the hepatocystic triangle, and 73.5% for the cystic plate criterion ^(11)^.

In addition, research on CV for surgical navigation has progressed. However, very few systems have been implemented in actual surgical settings. As of June 2021, the field of medical image diagnosis has witnessed significant advancements in the application of image recognition technology using AI. At that time, 343 AI-supported medical devices had received Food and Drug Administration approval, with over 70% related to radiology. Approximately half of all approved devices (48.5%) were intended for image diagnostic support, specifically computer-assisted diagnostic systems ^(12)^. In contrast, medical devices intended for computer-assisted surgery accounted for only 3.3% of the registrations. Among these, the software designed for preoperative planning using medical images constituted the majority, whereas there were no registrations for intraoperative navigation systems ^(12)^. In 2024, surgical image recognition support software has been approved by the Pharmaceuticals and Medical Devices Agency as a medical device ^(13)^. Approvals for other such medical devices are expected.

## Technical Evaluation of Endoscopic Surgery Predicting Surgical Outcomes

The postoperative use of AI includes predicting complications and outcomes using clinical information, including surgical records and surgical skill evaluations using surgical videos.

One study utilized the American College of Surgeons National Surgical Quality Improvement Program (ACS-NSQIP) database to identify patients who underwent liver, pancreatic, and colorectal surgeries between 2014 and 2016 and used decision tree models to predict 30-day postoperative complications. The algorithm had good predictive ability for any complication occurrence, with a C-statistic of 0.74, outperforming the traditional surgical risk calculator. The algorithm accurately predicted 13 of the 17 complications analyzed, particularly excelling in the prediction of stroke, wound dehiscence, cardiac arrest, and progressive renal failure ^(14)^.

**Figure 1.**
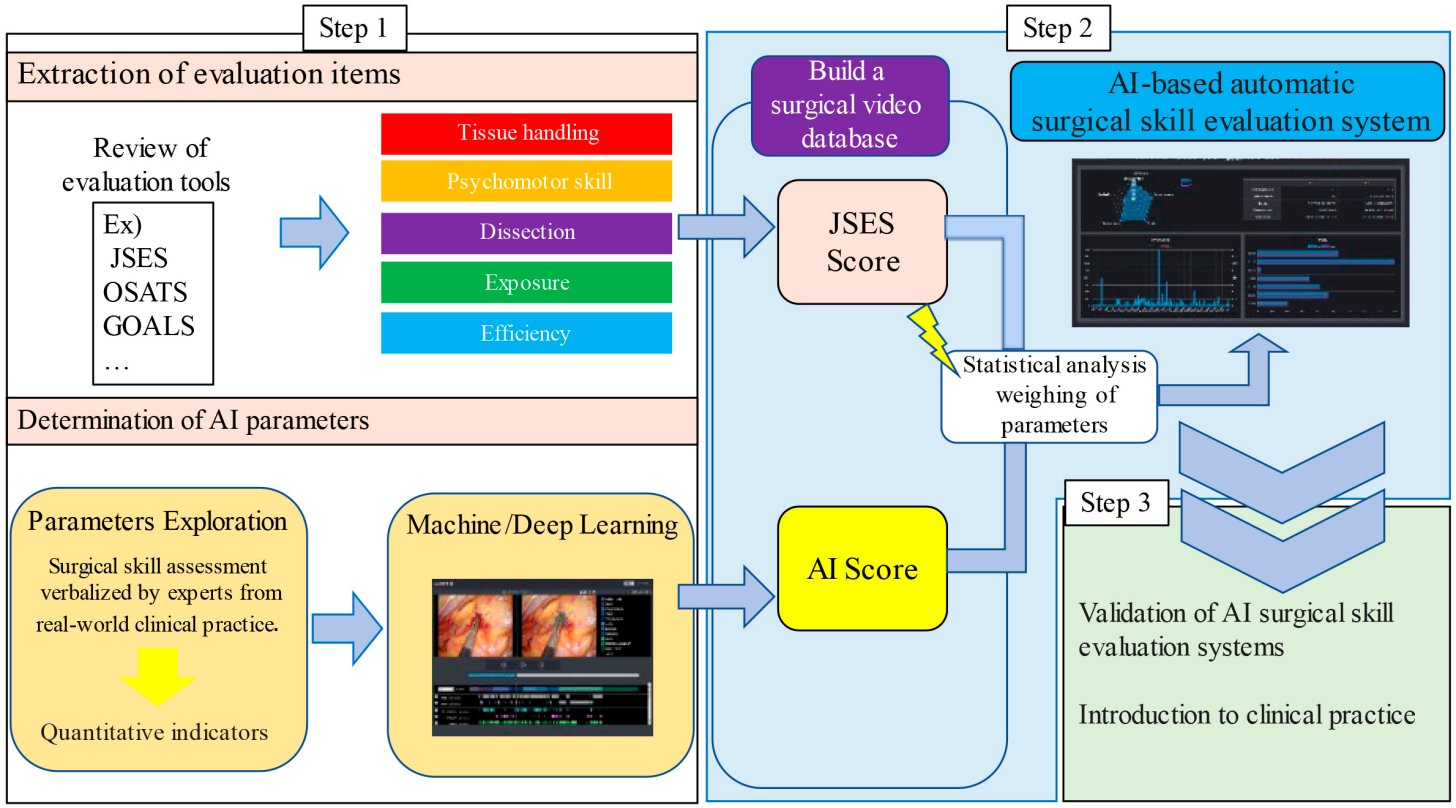
Development of an AI-based automatic surgical skill evaluation system for endoscopic surgery - project overview. JSES: Japan Society for Endoscopic Surgery. OSATS: Objective structured assessment of technical skills. GOALS: Globaloperative assessment of laparoscopic skills.

We developed an automated AI evaluation system for endoscopic surgery (Figure 1). We analyzed technical evaluation tools from domestic and international surgical skill assessment standards and various studies using qualitative research methods. We extracted 122 articles from PubMed, categorized them using an evaluation tool, and selected nine tools. From the descriptions and elements of these nine tools, 189 types of skill performance were extracted and organized into five comprehensive competencies: tissue handling, psychomotor skill, efficiency, dissection quality, and exposure quality ^(15)^.

Subsequently, an AI-based automatic surgical skill evaluation system was developed using surgical videos provided by the Japan Society for Endoscopic Surgery, utilizing expert technical evaluation results. Initially targeting laparoscopic sigmoidectomy, the surgical processes, instruments, and other relevant objects and actions in the surgical videos were analyzed and annotated. From the previously mentioned categories―tissue handling, psychomotor skills, efficiency, dissection quality, and exposure quality―several parameters were developed to automatically evaluate surgical skills using AI. One parameter was AI confidence score (AICS) for surgical phase recognition and the other was blood pixel count.

AICS is used to evaluate the standardization of surgical field development. Sixty videos of Lap-S with Endoscopic Surgical Skill Qualification System (ESSQS) scores of >75 points were used to construct a model. The output layer of the surgical phase recognition model utilizes the softmax function, which produces probabilities ranging from 0 to 1 and analyzes its similarity to the surgical field development, referred to as AICS. Additionally, the model could automatically screen for the low-scoring group with 93.3% specificity and 82.2% sensitivity and for the high-scoring group with 93.3% specificity and 86.7% sensitivity ^(16)^.

To automatically quantify the number of blood pixels in the surgical field, a machine learning model was developed using laparoscopic images to separate blood/nonblood pixels in the image using the red-green-blue values of each blood/nonblood pixel as supervised data. The number of pixels recognized as blood by the model was compared between the groups of surgeons with different tissue-handling skills evaluated using the ESSQS. The model’s overall accuracy was 85.7% having the lowest number of blood pixels in the group with the highest tissue-handling skills and the highest number in the novice surgeon group. The number of blood pixels measured by the model correlated significantly with the surgeon’s tissue-handling technique ^(17)^.

On the commercial side, services such as C-SATS ^(18)^ and Touch Surgery ^(19)^ promoted by surgical equipment manufacturers are expected to provide functions such as surgical video management, procedural analysis, and technical evaluation. These systems have already been introduced in Europe and the USA and are used by many doctors.

## Technical Hurdles and Future Expectations

**Figure 2.**
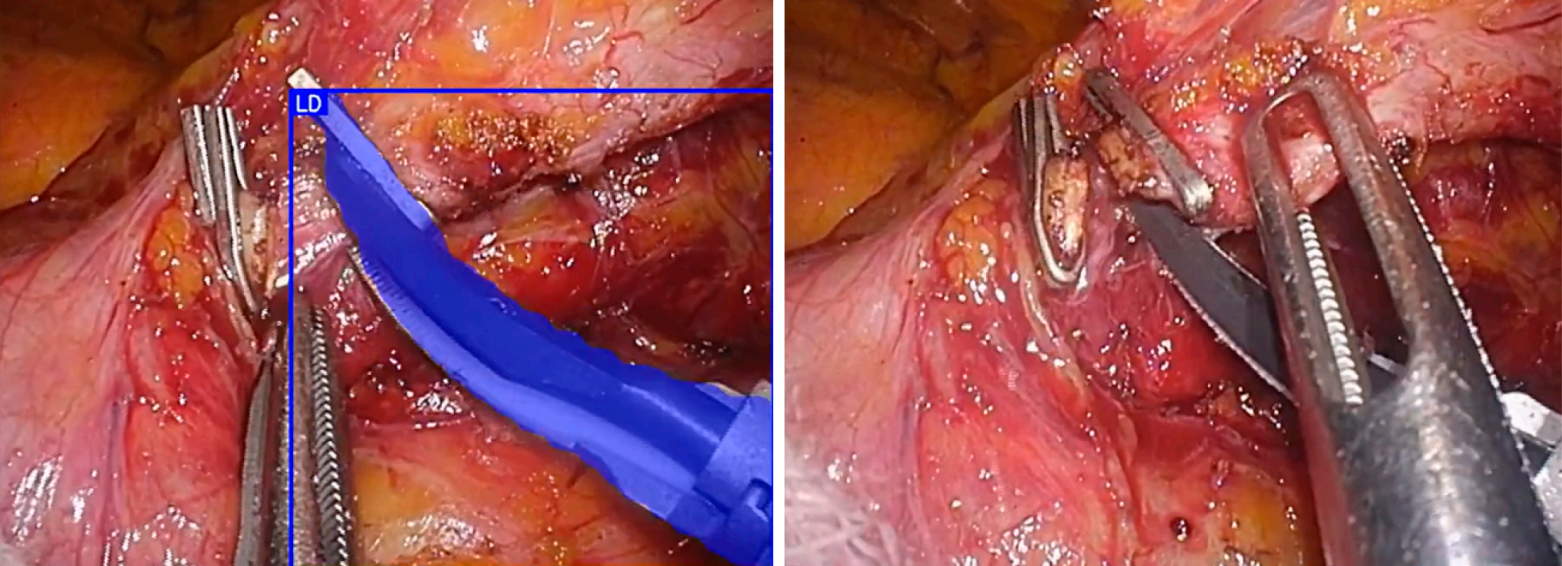
The laparoscopic energy device (LD), recognized by AI just previously, became unrecognized because its tip was covered.

The challenges faced by AI image recognition technology lie in recognition performance and generalizability. Current image recognition AI using surgical images faces performance issues owing to smoke, mist, blood, and fatty tissues obscuring the view during surgery. Performance declined significantly when objects were partially obscured (Figure 2). Although some issues can be addressed through engineering improvements, they cannot be resolved using existing methods that rely on image recognition.

Regarding generalizability, recognition performance declines when the camera type, field of view, and targeted organ differ. This is an unavoidable consequence of the inductive learning approach of AI, which learns from data and derives rules. One approach to improving the generalization performance involves increasing training data, thereby expanding the range that AI can handle. However, it is impractical to adopt this approach by simply adding one task because of the significant time, cost, and volume of data required. Therefore, technological innovations are required.

In recent years, the widespread adoption of surgical assistance robots has enabled the database and utilization of log data. This includes instrument manipulation by surgeons and the use of robotic arms and energy devices ^(20)^. In the era of laparoscopic surgery, this information can only be obtained by attaching sensors to the forceps and the surgeons’ bodies. However, in the era of robotic surgery, robots that function as forceps and sensors enable the continuous acquisition of surgical operation data without restrictions. Additionally, there is a movement to evaluate non-technical skills, such as situational awareness, decision-making, task management, leadership, communication, and teamwork ^(21)^. Through a multimodal approach that incorporates surgical videos, robot logs, and information from outside the surgical field, future laparoscopic surgeries can further visualize and quantify tacit knowledge. This has led to advancements not only in education but also in the development of new devices and systems and further automation of surgical procedures.

## Article Information

### Conflicts of Interest

None

### Acknowledgement

The Japan Agency for Medical Research and Development and the Japan Society for Endoscopic Surgery support the studies mentioned in the article.

### Author Contributions

Concept and design: Arakaki, Takenaka, Sasaki, Kitaguchi, Hasegawa, Takeshita, Takatsuki, Ito.

Drafting of the manuscript: Arakaki, Takenaka, Sasaki.

Critical manuscript revision for important intellectual content: Takenaka, Sasaki, Kitaguchi, Hasegawa, Takeshita, Takatsuki, Ito.

Supervision: Takenaka, Takeshita, Takatsuki, Ito.
